# Cohort profile: the Alma cohort of Swedish children born 2018–2023 – a prospective, register-based resource to study social determinants of health and Swedish child health services

**DOI:** 10.1136/bmjopen-2026-123576

**Published:** 2026-09-15

**Authors:** Anna Fäldt, Natalie Durbeej, Anton Dahlberg

**Affiliations:** 1 Child Health and Parenting (CHAP), Uppsala University, Uppsala, Sweden; 2 Child Health and Parenting (CHAP), Department of Public Health and Caring Sciences, Uppsala universitet Medicinska fakulteten, Uppsala, Sweden; 3 Folkvetenskap, Karlstads universitet Institutionen för hälsovetenskaper, Karlstad, Värmland County, Sweden

**Keywords:** Child, Health Equity, Health Services, Infant, Community child health

## Abstract

**Abstract:**

**Purpose:**

Social inequalities in child health persist even in high-income welfare states with universal child health services, yet large-scale longitudinal resources capturing early life exposures, family context and service delivery during the preschool years remain scarce. The Alma cohort was established to identify risk and protective factors for children’s health and development during the first 5 years of life, with a particular focus on social determinants of health and the delivery and impact of proportionate universalism within the Swedish child health services.

**Participants:**

The Alma cohort is a population-based register cohort comprising all Swedish children born between 2018 and 2023, together with their parents and siblings, totalling 2 169 423 unique individuals. Of these, 690 589 are index persons (children). Individual-level data are linked across multiple national registers using the unique Swedish personal identity number, including the Total Population Register, the Medical Birth Register, the National Patient Register, the Prescribed Drug Register, the Swedish Child Health Services Register (BHVQ), sociodemographic registers, registers on out-of-home placements and the Cause of Death Register. The cohort captures substantial variation in perinatal characteristics, family structure, socioeconomic conditions and geographical context. Data are retrieved in three waves up to 2030 to enable longitudinal follow-up from birth into school age.

**Findings to date:**

The cohort is broadly representative of Swedish children and families. Among index persons, 51.4% are boys and 48.6% are girls. Approximately 40% have at least one parent born outside Sweden, 63.5% have at least one parent with a college or university education and 78.0% reside in urban areas during their first year of life. The BHVQ subcohort, covering 458 724 index persons from 13 Swedish regions, provides data on child health and development and service delivery within the child health services. Several studies are currently ongoing, addressing topics such as language screening, enhanced support from child health services, parental mental disorders, out-of-home care and the consequences of parental loss.

**Future plans:**

Planned data extractions will extend follow-up through early school age, including linkage to the National School Register for data on school performance. The cohort is open to collaboration with researchers interested in child health, service evaluation and policy-relevant analyses.

**STRENGTHS AND LIMITATIONS OF THIS STUDY**

- The Alma cohort links individual-level data across nine national registers using the unique Swedish personal identity number, enabling comprehensive longitudinal follow-up of children, parents and siblings from birth through early school age without attrition.
- The population-based design captures all Swedish children born between 2018 and 2023, comprising 690 589 index persons embedded within a total study population of over 2.1 million individuals, providing sufficient statistical power for analyses of rare exposures and outcomes.
- The inclusion of the Swedish Child Health Services Register (BHVQ) adds a methodologically distinctive dimension, enabling direct linkage between child health and developmental data and service delivery variables during the preschool years, a period seldom captured in population-based register research.
- The retrospective register-based design restricts available variables to those collected for administrative or clinical purposes, which may limit measurement precision and introduces a risk of misclassification for some exposures and outcomes.
- The BHVQ currently covers 13 of Sweden’s 21 regions, meaning that analyses incorporating child health services data must be conducted as subcohort analyses and may not be fully representative of the national population with respect to geographical distribution.

## Introduction

 Children in high-income countries, including the Nordic welfare states, generally enjoy good health and access to universal services. Yet childhood morbidity, mental health problems, obesity and developmental difficulties remain important public health concerns, and these outcomes are disproportionally distributed across the population.^1^ A large body of European and Nordic research shows persistent social gradients in child and adolescent health by parental education, income, family structure and migrant background, despite extensive welfare provisions and universal child health services.^2^ These inequalities emerge early in life and tend to accumulate across childhood and adolescence.^3^ It is therefore crucial to monitor and support children’s health and development within universal systems, and to understand how policies and services can reduce—rather than reproduce—existing inequalities.^4^ Further, knowledge about risk factors for early health and developmental deviations remains limited. Most studies examine children later, typically during middle childhood.

Several socioeconomic status factors are associated with young children’s health and development. For instance, low parental income, low parental education level, parental unemployment, single marital status and parental immigrant status are associated with both mental health problems and somatic problems.^5–8^ A parental college degree or higher, steady employment, adequate access to education, health services and food is protective for the child’s health.^9 10^ Exposure to green space is associated with perinatal health, physical activity and respiratory health.^11^

Further, circumstances during both the prenatal and perinatal periods affect the child’s future health and development.^12^ Prenatal maternal factors include preconception body mass index, age, depression and anxiety.^13^

Adversity and stressful life events impact children’s health outcomes. Children exposed to siblings with chronic illnesses have higher levels of mental health problems compared with children with healthy siblings.^14^ Similarly, parental illness and death are associated with child mental health and somatic problems, as well as increased risk of childhood injuries.^15 16^ Children placed in foster care have elevated rates of subsequent mental health problems, physical health problems, higher suicide risk and less participation in Child Health Services during the preschool period than children of the general population.^17–19^

There is a lack of studies that follow children during the formative preschool years with regard to identified protective and risk factors, as well as how child health services influence their development and health. Longitudinal studies at the population level are necessary.

In Sweden, the child health services, established in 1937, systematically follow children during this period and monitor their development and health. The services are publicly funded and provide health promotion services to nearly all Swedish children from birth to age 5. The services are structured within a national programme to promote health, monitor development and provide parental support. Universal healthcare visits are most intense during the first year of life, followed by visits at 1½, 2½, 3, 4 and 5 years of age.^20^ The primary focus of the child health services is on universal services, including health monitoring, parenting support, continuous growth assessments, somatic and psychomotor surveillance and assessments of general, behavioural, language and communication development.

To address the research gap described above, we established the Alma cohort. The cohort was designed to identify risk factors in children and their families using reliable and valid data sources, with the explicit purpose of assessing social determinants of health in the Swedish context and examining the delivery and impact of proportionate universalism within Swedish child health services. Through linking several Swedish registers, the Alma cohort provides unique opportunities to study a multitude of factors that influence children’s health, health trends during the first 5 years and later developmental and health outcomes. The current paper describes the set-up of the Alma cohort, which includes Swedish children aged 0–5 years.

## Cohort description

### Data sources and domains

All individuals of the cohort are retrieved from the Swedish Total Population Register (TPR), containing data on life events including birth, death, name change, marital status, family relationships and migration within, to and from Sweden.^21^ Index persons with data from other registers but not from TPR are excluded.

The Swedish Child Health Services Register (BHVQ) delivers data on the index persons’ health and development as well as on service delivery within the Swedish child health services. This register includes data from 13 Swedish Regions, with data automatically extracted from the Child Health Services Records.^22^

The Swedish National Patient Register (SNPR) is used to collect data on somatic and psychiatric diagnoses among index persons, siblings and parents, and healthcare utilisation. The SNPR includes dates of inpatient hospital care, visits to specialised outpatient clinics and diagnoses according to International Classification of Diseases, 10th Revision (ICD-10) codes. The register was established in 1964, became nationwide with respect to inpatient treatment in 1987, and with respect to non-private specialised outpatient treatment in 2001. Overall, the register has demonstrated high validity and suitability for large-scale population research.^23^

The Prescribed Drug Register (PDR) delivers data on prescribed medication to index persons and parents. The PDR was launched in 2005 and contains information on all prescriptions dispensed at pharmacies to Swedish inhabitants. All drugs are classified according to the WHO Anatomical Therapeutic Chemical classification. The PDR has a high coverage of the Swedish population (>99.7%) and has been found useful for epidemiological research.^24^

Data on mothers and newborns are collected from the Medical Birth Register, established in 1973. This register provides data on pregnancies, deliveries and newborns. The register covers 98% of all births in Sweden including information on the mother’s age, parity, the date of the child’s birth, various maternal characteristics and pregnancy outcomes.^25^

The National Register of Measures for Children and Young persons is used to collect information on out-of-home placements among index persons. This register was established in 1994 and includes statistics on placements, including information on guardians, type of intervention, dates of decision, placement and termination and form of placement. In addition, it includes specifications of placements under the Social Services Act, as well as under the Care of Young Persons (Special Provisions) Act.

We also collect sociodemographic data from the Swedish Longitudinal Integrated Database for Health Insurance and Labour Market Studies (LISA, Longitudinell Integrationsdatabas för Sjukförsäkrings- och Arbetsmarknadsstudier) on various sociodemographic domains, namely parental education, occupation, income, sick leave, disability pension, unemployment benefits, disposable income and social welfare payments in parents. The LISA database was launched in 1990 and has high coverage in the various sociodemographic domains.^26^

Geographical and area information data is collected as demographic statistical areas,^27^ which is a small-area classification system developed by Statistics Sweden.

Finally, the Cause of Death Register (CDR) is used to collect data on deaths in index persons and parents. The CDR includes all deaths of Swedish inhabitants reported to the Swedish tax authority since 1997, according to ICD-10 codes as listed on the death certificate by the responsible physician. The register has been found to have large completeness, high quality and suitability for research purposes.^28^

**Figure 1:**
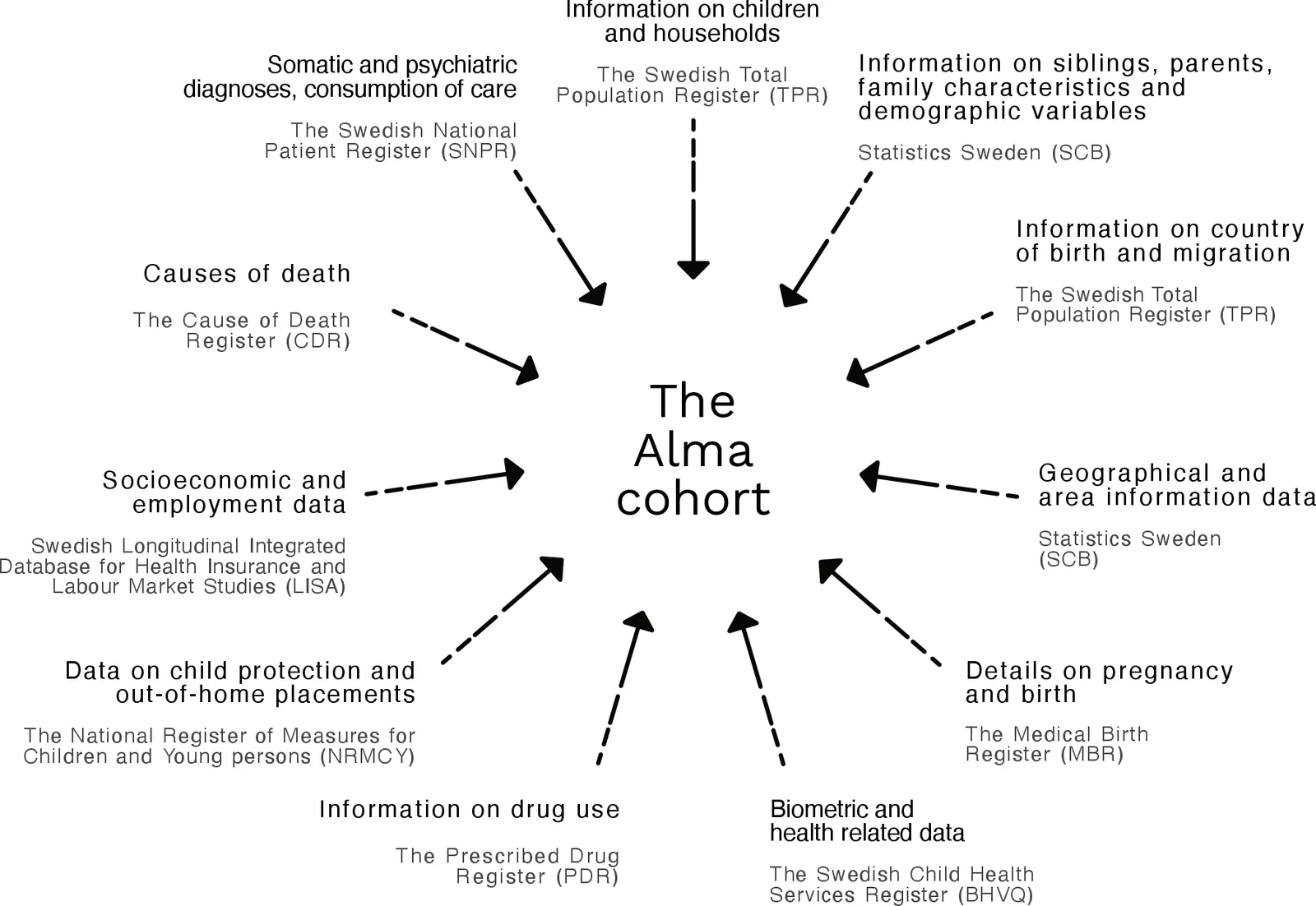
National registers included in the Alma cohort and their linkage to index persons, parents and siblings.

All the registers are specified in figure 1.

### Record linkage

**Figure 2:**
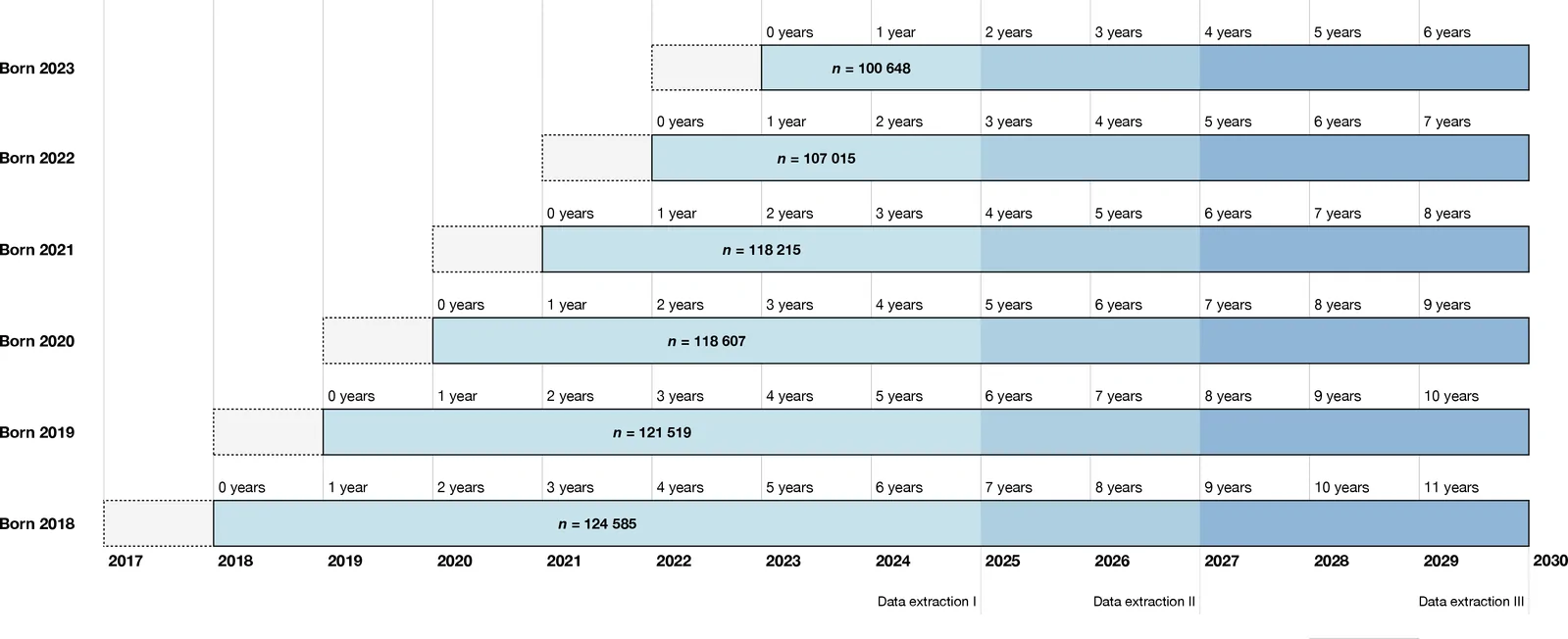
Birth cohorts of index persons and timing of retrospective data extractions in the Alma cohort.

Record linkage across registers is enabled through the unique Swedish personal identity number assigned to all residents. Most variables are specified with an exact date, such as diagnoses, prescribed medication and visits to child health services. Data from TPR and the LISA database are registered across a given calendar year. Data are retrieved in three waves until 2030, to follow the health and development across time (figure 2). In the last data retrieval, we will also collect data from the National School Register on school performance as an outcome. There are also ongoing plans to add data from speech and language pathology services at two timepoints.

### Public and patient involvement

No public and patient involvement (PPI) was included in the design of this cohort profile; however, PPI is planned and initiated for future studies.

## Findings to date

### Study population

The total cohort comprises 2 169 423 unique individuals. Of these, 690 589 are index persons (children), of whom 323 699 also appear as siblings. In total, there are 750 912 siblings, including 4661 who are also parents. The cohort further includes 1 055 157 parents, encompassing biological parents, adoptive parents and legal guardians. Figure 2 provides a visual overview of the number of index persons born each year and the timing of retrospective data extractions. The number of children born each year diminished over time, a pattern that mirrors the declining birth rates in Sweden and does not indicate systematic changes in the underlying register data.

Table 1 presents the distribution of sociodemographic and perinatal characteristics among the index persons in the Alma cohort, including parental country of birth, educational level and geographical location during the child’s first year of life.

**Table 1:** Sociodemographic and perinatal characteristics of index persons and their parents in the Alma cohort

Variables	n	%
Total (n=690 589)		
Boy	354 781	51.4
Girl	335 808	48.6
Birth year (n=690 589)		
2018	124 585	18.0
2019	121 519	17.6
2020	118 607	17.2
2021	118 215	17.1
2022	107 015	15.5
2023	100 648	14.6
Perinatal characteristics (n=655 564)		
Multiple birth	17 071	2.6
Preterm (<37 weeks)	34 542	5.3
Child’s foreign or Swedish background (n=690 589)		
Born outside Sweden	26 102	3.8
Born in Sweden, both parents born outside Sweden	152 643	22.1
Born in Sweden, one parent born outside Sweden	99 114	14.4
Born in Sweden, both parents born in Sweden	412 730	59.8
Highest level of parental education at child’s first year of life (n=690 589)		
College or university	438 572	63.5
Upper secondary school or lower	239 745	34.7
Data missing	12 272	1.8
Geographical location at child’s first year of life (n=690 589)		
Rural areas (A)	94 931	13.7
Semiurban areas (B)	56 244	8.1
Urban areas (C)	538 942	78.0
Missing data	472	0.1
Child household density during the first year of life (n=690 589)		
Overcrowded <10 m² per person	18 461	2.7
Not overcrowded	653 771	94.7
Missing data	18 357	2.7

Percentages may not sum to 100 due to rounding.

Preliminary results indicate gender differences in causes of parental death, as well as a social gradient, where lower income and education levels were associated with higher risk of parental death (submitted manuscript). Further, weight for gestational age has been assessed in relation to language screening results at the child health services. Being both small and large for gestational age were associated with failing language screening.^29^

### Available data from the BHVQ

As not all Swedish regions currently submit data to the BHVQ, data for the entire Alma cohort is not available. The BHVQ cohort includes a total of 458 724 index persons and is broadly representative with respect to most sociodemographic variables. However, the distribution across birth years differs from that of the Alma cohort, with a smaller proportion born in 2018 (12.9%) compared with other years. The cohort also has a higher proportion of index persons with one parent born abroad (38.4%). As BHVQ includes data from the three metropolitan regions, the geographical distribution is affected: 12.5% of the index persons were born in rural areas and 79.8% in urban areas. Analyses using BHVQ data will therefore typically be conducted as sub-cohort analyses.

## Ongoing and planned studies

Several studies are ongoing or planned within the Alma cohort. These include investigations of how home visits, individual visits and team visits relate to risk factors defined in the Swedish national programme for child health services, as well as studies of language screening and subsequent referrals, with particular focus on children with parents born outside Sweden. Other studies address whether enhanced support from child health services improves children’s subsequent health and include a health economic evaluation of the Swedish child health services. Further work examines the impact of parental mental disorders on child health and development, the role of socioeconomic factors for development, health and healthcare utilisation, and the health and developmental trajectories of children in out-of-home care. Additional studies investigate associations between perinatal circumstances and later development and health, the influence of parents’ and siblings’ chronic conditions, and the effects of living conditions on health, development and care consumption. Finally, the cohort is used to study the consequences of parental loss during early childhood, including differences by cause of death.

The choice of statistical method will be tailored to the specific research question of each substudy, but analyses will generally draw on regression-based approaches suited to register data, including linear, logistic, Poisson/negative binomial and Cox proportional hazards regression, depending on outcome type. As several outcomes are hierarchically structured (children nested within families, and families within regions or BHVQ catchment areas) mixed-effects models will be used where appropriate to account for clustering.

As an illustrative example, the planned study examining whether enhanced support from child health services improves children’s subsequent health will need to address confounding by indication, since children receiving enhanced support are likely to differ systematically from those who do not (for example, in perinatal risk, socioeconomic circumstances or somatic diagnoses). We plan to handle this through covariate adjustment guided by directed acyclic graphs. The accompanying health economic evaluation will apply standard methods for register-based economic evaluation, estimating healthcare and societal costs (such as inpatient and outpatient care, prescribed medication) associated with enhanced support relative to standard care.

## Strengths and limitations

The Alma cohort has several important strengths. First, its size and national coverage are considerable: the cohort includes more than 2 million individuals, comprising index persons, parents and siblings. This provides a unique possibility to study child health and development in a population-based setting with sufficient statistical power also for relatively rare exposures and outcomes. The design ensures that the study population is broadly representative of Swedish children and families, although some sociodemographic groups may be slightly under-represented.

A second strength is the richness of data and the multiple perspectives that are included. Analyses are possible not only for the index person, but also for parents and siblings, enabling investigation of family and intergenerational factors. The cohort draws on several high-quality national registers, most of which have been thoroughly evaluated for validity and coverage. This contributes to high data quality and allows for the study of health, sociodemographic and service-related factors in detail. The possibility of continuous record linkage on the individual level, using the unique Swedish personal identity number, further strengthens the cohort.

A further strength is the wide applicability of the cohort. The Alma cohort can be used for studies of perinatal and early life exposures, the evaluation of child health services and the study of a broad range of somatic and mental health outcomes, both in childhood and later in life. The inclusion of the BHVQ adds a unique dimension, as it provides information on service delivery and child development and health during the preschool years, a period often under-represented in longitudinal population-based research. To the best of our knowledge, this is the first population cohort in Sweden that includes data from the child health services and thereby enables the study of early child development and child health monitoring.

Nevertheless, some limitations should be noted. The retrospective design implies that exposure and outcome variables are retrieved from existing registers, which restricts the range of variables to those already collected for administrative or clinical purposes. This limits flexibility in measurement and may introduce misclassification. Attrition over time is not an issue in the traditional sense, since register-based follow-up is virtually complete, but data availability may vary between registers and regions. For example, the BHVQ currently does not cover all regions in Sweden, which may affect representativeness. Another limitation is that the SNPR only includes diagnoses by medical doctors, thereby excluding diagnoses mainly by other professionals, such as speech and language therapists, physiotherapists and psychologists. Finally, generalisability is a consideration. While the cohort is highly representative of the Swedish population, caution is needed when extrapolating findings to other countries with different healthcare systems, social policies, or population structures. Within Sweden, however, the results should be broadly generalisable at national level.

## Collaboration

Several collaborations between different Swedish research groups are ongoing. The Alma cohort also enables international comparisons with registry-based resources from other countries, in which childhood policies across different countries, welfare-systems and contexts can be studied, as has been done in previous studies.^30–32^ The cohort is open to collaboration with researchers interested in child health, child health service evaluation and policy-relevant analyses. Enquiries may be directed to the principal investigator, Dr Anna Fäldt (Anna.Faldt@uu.se) and Anton Dahlberg (anton.dahlberg@uu.se).

## Data Availability

All data relevant to the study are included in the article or uploaded as supplementary information.
