# Supplementary material for: Cohort profile: the Alma cohort of Swedish children born 2018–2023 – a prospective, register-based resource to study social determinants of health and Swedish child health services
Source: BMJ Open. 2026 Sep 15;16(9):e123576. doi: 10.1136/bmjopen-2026-123576 (PMC13583863; doi:10.1136/bmjopen-2026-123576)
Supplement: Supplementary file 1 [file bmjopen-2026-123576.reviewer_comments.pdf]

PEER REVIEW HISTORY

BMJ Open publishes all reviews undertaken for accepted manuscripts. Reviewers are asked to complete a checklist review form and are provided with free text boxes to elaborate on their assessment. These free text comments are reproduced below.

ARTICLE DETAILS

Title (Provisional)

Cohort profile: The Alma cohort of Swedish children born 2018 to 2023—a prospective, register-based resource to study social determinants of health and Swedish child health services

Authors

Fäldt, Anna; Durbeej, Natalie; Dahlberg, Anton

VERSION 1 - REVIEW

**Reviewer**	**1**
**Name**	**Spencer, Nick**
**Affiliation**	**University of Warwick, School of Health and Social Studies**
**Date**	**04-Aug-2026**
**COI**	**None**

In reviewing this cohort profile, I'll make some general observations before addressing the specific questions posed by the BMJ Open editors.

General observations:

This is the latest example of the potential power of longitudinal research based on reliable population-based administrative databases that can be linked to index 'cases'. The National Birth Registers in Nordic in Sweden, Denmark and Norway have proved a rich source of data on children's health and wellbeing and the impact of their social circumstances and socioeconomic policies across the early life course. The Alma cohort extends this work using a range of linked data sources to focus on early childhood. The addition of the BHVQ, despite the limitations acknowledged by the authors, offers an additional opportunity to shed new light on a long-standing debate about the efficacy of regular surveillance in early childhood. I enthusiastically endorse the establishment of this cohort while suggesting some additions below that I think strengthen it.

Editor's questions:

● Are the specific items in the abstract, pertinent to cohort profiles, clear and comprehensive (Findings to date, Future plans)? YES

● Does the introduction include a clear and persuasive rationale? Partially, but there should be more discussion on what this cohort adds to existing birth cohorts and existing work using the National Birth Registers especially in Sweden

● Is the cohort well described? YES – figure 1 presents a comprehensive list of the linked registers included in the cohort

● Cohort profiles should include a section on Findings to date, covering a short explanation of the most notable results from the cohort so far; summarising rather than exhaustively presenting findings. The section on Results To Date is limited to data on the numbers of index children and their families/siblings and their sociodemographic characteristics

● Is the Collaboration section adequately outlining data availability and access and what kind of collaborations are encouraged? PARTIAL – I think collaboration should specify potential international collaborations allowing comparative analysis of early childhood policies and interventions. See examples ([1],[2],[3]) of these collaborations that show different childhood outcomes by country and throw light on the impact of different early childhood policies

Additional comment on section 'Ongoing and planned studies':

I think the authors should include more on proposed analysis/stats methods. For example, they could include a brief reference to how they plan to use the BHVQ data to, as they state in the paper (p.13, lines 243-5) "address whether enhanced support from child health services improves children's subsequent health and include a health economic evaluation of the Swedish child health services"

Minor issue:

Incomplete ref: Pearce, A.(2020) Reducing inequities in health across the life-course: early years, childhood and adolescence.

References:

[1] Zylbersztejn A, Gilbert R, Hjern A, Wijlaars L, Hardelid P. Child mortality in England compared with Sweden: a birth cohort study. Lancet. 2018 May 19;391(10134):2008-2018. doi: 10.1016/S0140-6736(18)30670-6.

[2] White PA, Awad YA, Gauvin L, Spencer NJ, McGrath JJ, Clifford SA, Nikiema B, Yang-Huang J, Goldhaber-Fiebert JD, Markham W, Mensah FK, van Grieken A, Raat H, Jaddoe VWV, Ludvigsson J, Faresjö T; EPOCH Collaborative Group. Household income and maternal education in early childhood and risk of overweight and obesity in late childhood: Findings from seven birth cohort studies in six high-income countries. Int J Obes (Lond). 2022 Sep;46(9):1703-1711. doi: 10.1038/s41366-022-01171-7

[3] Nikiema B, Spencer N, Seguin L. Poverty and Chronic Illness in Early Childhood: A Comparison Between the United Kingdom and Quebec. Pediatrics 2010; 125 (3): e499–e507. Doi: www.pediatrics.org/cgi/doi/10.1542/peds.2009-0701

**Reviewer**	**2**
**Name**	**DALAI, RICHIE**
**Affiliation**	**All India Institute of Medical Sciences - Patna, Neonatology**
**Date**	**05-Aug-2026**
**COI**	**None**

The authors need to mention the trial registration numbers of the associated trials of the cohort, and at least their research questions if these studies are yet to be published.

VERSION 1 - AUTHOR RESPONSE

Reviewer: 1

Comments to the Authors:
In reviewing this cohort profile, I'll make some general observations before addressing the specific questions posed by the BMJ Open editors.

General observations:
This is the latest example of the potential power of longitudinal research based on reliable population-based administrative databases that can be linked to index 'cases'. The National Birth Registers in Nordic in Sweden, Denmark and Norway have proved a rich source of data on children's health and wellbeing and the impact of their social circumstances and socioeconomic policies across the early life course. The Alma cohort extends this work using a range of linked data sources to focus on early childhood. The addition of the BHVQ, despite the limitations acknowledged by the authors, offers an additional opportunity to shed new light on a long-standing debate about the efficacy of regular surveillance in early childhood. I enthusiastically endorse the establishment of this cohort while suggesting some additions below that I think strengthen it.

Our response: We are grateful for the reviewer's positive assessment of our work and for the thoughtful suggestions provided, which we address in detail below.

Does the introduction include a clear and persuasive rationale? Partially, but there should be more discussion on what this cohort adds to existing birth cohorts and existing work using the National Birth Registers especially in Sweden

Our response: Thank you for this suggestion. We have added discussion on this in the Introduction section (p. 7, lines 124–127). This is also discussed further in the Strengths and limitations section (p. 15, lines 278–280).

Cohort profiles should include a section on Findings to date, covering a short explanation of the most notable results from the cohort so far; summarising rather than exhaustively presenting findings. The section on Results To Date is limited to data on the numbers of index children and their families/siblings and their sociodemographic characteristics

Our response: Thank you for asking for additional information. We have added preliminary results from a submitted manuscript focusing on children who have lost a parent during the first five years of life, and a recently published paper on SGA/LGA in relation to language screening results (p. 11, lines 208–213).

Is the Collaboration section adequately outlining data availability and access and what kind of collaborations are encouraged? PARTIAL – I think collaboration should specify potential international collaborations allowing comparative analysis of early childhood policies and interventions. See examples ([1],[2],[3]) of these collaborations that show different childhood outcomes by country and throw light on the impact of different early childhood policies

Our response: Good suggestion! We have added this to the Collaboration section (p. 15, lines 296–299).

Additional comment on section 'Ongoing and planned studies':
I think the authors should include more on proposed analysis/stats methods. For example, they could include a brief reference to how they plan to use the BHVQ data to, as they state in the paper (p.13, lines 243-5) "address whether enhanced support from child health services improves children's subsequent health and include a health economic evaluation of the Swedish child health services"

Our response: Thank you for this suggestion. We have added a paragraph at the end of the *Ongoing and planned studies* section, where we discuss proposed approaches to analyse the data (p. 13, lines 240–255).

Minor issue:
Incomplete ref: Pearce, A.(2020) Reducing inequities in health across the life-course: early years, childhood and adolescence.

Our response: Thank you for pointing this out. We have updated this reference. In addition, the references suggested by the reviewer have been incorporated into the manuscript.

Reviewer: 2

Dr. RICHIE DALAI, All India Institute of Medical Sciences - Patna
Comments to the Authors:
The authors need to mention the trial registration numbers of the associated trials of the cohort, and at least their research questions if these studies are yet to be published.

Our response: We thank the reviewer for this comment. All studies within the cohort are observational, register-based studies rather than trials involving researcher-allocated interventions, and trial registration is therefore not applicable
